# Effects of Adjuvants on the Immunogenicity and Efficacy of a Zika Virus Envelope Domain III Subunit Vaccine

**DOI:** 10.3390/vaccines7040161

**Published:** 2019-10-27

**Authors:** Xinyi Wang, Wanbo Tai, Xiaolu Zhang, Yusen Zhou, Lanying Du, Chuanlai Shen

**Affiliations:** 1 Department of Microbiology and Immunology, Medical School, Southeast University, Nanjing 210009, China; 2 Lindsley F. Kimball Research Institute, New York Blood Center, New York, NY 10065, USA; 3 State Key Laboratory of Pathogen and Biosecurity, Beijing Institute of Microbiology and Epidemiology, Beijing 100071, China; 4 Institute of Medical and Pharmaceutical Sciences, Zhengzhou University, Zhengzhou 450001, China

**Keywords:** flavivirus, Zika virus, envelope domain III, immune response, protective efficacy, adjuvants, subunit vaccine

## Abstract

Zika virus (ZIKV), a mosquito-borne flavivirus, has attracted global attention due to its close association with congenital Zika syndrome and neurological diseases, and transmission through additional routes, such as sexual contact. Currently there are no vaccines approved for ZIKV, and thus, there is an urgent need to develop an effective and safe ZIKV vaccine. Domain III (DIII) of the ZIKV envelope (E) protein is an important vaccine target, and a vaccine developed using a mutant DIII of E (EDIII) protein protects adult and pregnant mice, and unborn offspring, against ZIKV infection. Here, we have used immunocompetent BALB/c mice treated with anti-interferon-α/β receptor 1 (Ifnar1) antibodies to investigate whether three adjuvants (aluminum (Alum), monophosphoryl lipid A (MPL), and MF59), either alone or in combination, could improve the efficacy of this EDIII subunit vaccine. Our data show that, although vaccine formulated with a single adjuvant induced a specific antibody and cellular immune response, and reduced viral load in mice challenged with ZIKV, the combination of Alum and MPL adjuvants led to a more robust and balanced immune response, stronger neutralizing activity against three recent ZIKV human strains, and greater protection against a high-dose ZIKV challenge. Particularly, the combination of Alum with MPL significantly reduced viral titers and viral RNA copy numbers in sera and tissues, including the male reproductive organs. Overall, this study has identified the combination of Alum and MPL as the most effective adjuvant for ZIKV EDIII subunit vaccines, and it has important implications for subunit vaccines against other enveloped viruses, including non-ZIKV flaviviruses.

## 1. Introduction

Zika virus (ZIKV) is a mosquito-borne flavivirus first isolated from a rhesus macaque in Uganda in 1947 [1]. Most ZIKV infections are asymptomatic or show mild clinical symptoms, such as fever, headache, or rash. However, some adults infected with ZIKV present with Guillain–Barré syndrome (GBS), a disorder of the nervous system that results in muscle weakness and/or paralysis [2,3]. In addition, infection during pregnancy may cause congenital Zika syndrome (CZS), which is associated with microcephaly, fetal demise, and other congenital disorders [4,5,6]. Thus, ZIKV has emerged as a global health threat and there is an urgent requirement for an effective vaccine to combat ZIKV-associated diseases.

The ZIKV genome encodes a single polyprotein, which is processed into three structural proteins (capsid (C), precursor of membrane/membrane (prM/M), and envelope (E)) and seven non-structural proteins (NS1, NS2A, NS2B, NS3, NS4A, NS4B, and NS5) [7,8,9]. The E protein has important roles in infection and pathogenesis, is involved in virus binding to target cells and membrane fusion, and also serves as an important vaccine target [7,8]. The E protein is a transmembrane protein and consists of three domains termed I, II, and III (DI–DIII), a fusion loop (FL), and a stalk region (S) [8]. The DI–DII region of ZIKV E protein is a target for cross-reactive antibodies with other flaviviruses, such as dengue virus (DENV) and West Nile virus (WNV), whereas the DIII domain induces ZIKV-specific antibodies [10,11,12], and therefore, is a key target for ZIKV vaccine development. ZIKV vaccine candidates under development include inactivated virus, live attenuated virus, DNA, mRNA, viral vectors, virus-like particles (VLPs), and viral proteins (or subunit vaccines) [13,14,15,16]. Although many have advanced clinical trials and been tested for immunogenicity and/or levels of protection [17,18,19], none are approved for use in humans. Thus, continuous efforts are required to develop safe and efficacious ZIKV vaccines.

Subunit vaccines do not contain live virus or normally elicit adverse side effects and are safe for use in humans including pregnant women and unborn offspring [20,21]. However, subunit vaccines often suffer from low immunogenicity, although this may be improved by adjuvants. Adjuvants are important components of vaccines as they enhance the immunogenicity of vaccine antigens by boosting the quality and duration of the humoral and/or cellular immune response. Aluminum salts (Alum) are approved for use as adjuvants for human vaccines in the U.S. They are also key components of vaccines for a number of viruses including Hepatitis A virus (HAV), Hepatitis B virus (HBV), and human Papilloma virus (HPV) [22,23]. The Alum adjuvants most commonly used in licensed vaccines are Alum hydroxide and Alum phosphate [24]. Monophosphoryl lipid A (MPL), a non-toxic derivative of lipopolysaccharide (LPS) and a toll-like receptor (TLR) agonist, can also be used as an adjuvant for human vaccines [25]. In addition, AS04 (a combination of Alum and MPL) is used in vaccines for HBV and HPV [23], while MF59 (an oil-in-water emulsion) has been approved for use with conjugated vaccines for a number of viruses, including influenza virus, herpes simplex virus (HSV), and human immunodeficiency virus (HIV) [22,23].

Previously, we have shown that a ZIKV E protein DIII-based subunit vaccine, fused with the Fc region of human IgG, induced specific neutralizing antibodies against divergent ZIKV strains [26]. Furthermore, masking a non-neutralizing epitope (centered on residue 375) of this protein by a glycan probe (DIII of E (EDIII)) enhanced the neutralizing antibody response, and increased protection of adult and pregnant mice against ZIKV challenge without any impact on fetal development [27]. Here, using this EDIII-based subunit vaccine, we compared the adjuvanticity of MF59, Alum, MPL, or Alum + MPL combination (refer to AS04), in a hope to identify an adjuvant or adjuvant combination that is able to promote the EDIII-based ZIKV vaccine to induce a balanced immune response and potent protection against ZIKV infection. Our results show that a more balanced (Th1/Th2) immune response is generated and that the immunogenicity and the ability to protect against ZIKV infection are significantly enhanced when both Alum and MPL are used in the same vaccine formulation. This study has identified the combination of Alum with MPL as an effective adjuvant formulation for ZIKV EDIII-based subunit vaccines.

## 2. Materials and Methods

### 2.1. Animals

Four-to-five-week-old male BALB/c mice were used in the study. The animal studies were carried out in strict accordance with the recommendations in the Guide for the Care and Use of Laboratory Animals, National Research Council Committee [28]. The protocols have been approved by the Institutional Animal Care and Use Committee (IACUC) of the New York Blood Center (permit numbers: 344 and 345).

### 2.2. Expression and Purification of a Recombinant ZIKV EDIII Protein

This protein was prepared as previously described [27]. Briefly, ZIKV DIII of E protein containing M375N and E377T mutations at residues 375 and 377 of E protein (hereinafter EDIII) was constructed using a mutagenesis kit (Thermo Fisher Scientific, Waltham, MA, USA) and a *ZIKV* wild-type plasmid expressing residues 298–409 (DIII) of E protein and a C-terminal Fc tag of human (hFc) IgG1 [26,27]. The recombinant EDIII protein was transiently expressed in the culture supernatant of 293T cells, and purified by protein A affinity chromatography (GE Healthcare, Chicago, IL, USA).

### 2.3. Mouse Immunization

The above purified ZIKV EDIII protein was used to immunize mice in the presence or absence of various adjuvants as previously described [27]. Briefly, mice were intramuscularly (i.m., 100 μL/mouse) immunized with EDIII protein (10 μg/mouse) and one of the following adjuvant(s): Alum (i.e., aluminum hydroxide, 500 μg/mouse, InvivoGen, San Diego, CA, USA), MPL (10 μg/mouse, InvivoGen), Alum (500 μg/mouse) + MPL (10 μg/mouse), or MF59 (50 μL/mouse) [29]. Mice injected with EDIII protein or phosphate-buffered saline (PBS) only were included as controls. The immunized mice were boosted once with the same immunogens at three weeks, and sera were collected at 7 days post-last dose to detect antibody responses and neutralizing antibodies, as described below.

### 2.4. ELISA

ZIKV E, EDIII, or hFc-specific antibodies in immunized mouse sera were analyzed by ELISA as previously described [30]. Briefly, ELISA plates were coated with ZIKV EDIII protein, ZIKV full-length E protein with a His_6_ tag (Aviva Systems Biology, San Diego, CA, USA), or a C-terminal hFc-fused control protein containing a receptor-binding domain (i.e., RBD-Fc) of Middle East respiratory syndrome coronavirus (MERS-CoV) spike protein [31] (1 μg/mL) overnight at 4 °C, and blocked with 2% fat-free milk in PBST (PBS containing tween-20) at 37 °C for 2 h. The plates were washed with PBST for 3 times, and sequentially incubated with serial dilutions of mouse sera and horseradish peroxidase (HRP)-conjugated anti-mouse IgG (1:5000) or IgG-Fab (1:3000) (for anti-ZIKV-E or anti-hFc antibodies), IgG1 (1:5000), or IgG2a (1:2000) antibodies (Thermo Fisher Scientific) at 37 °C for 1 h. The reaction was visualized after addition of 3,3′,5,5′-tetramethylbenzidine substrate (Sigma, St. Louis, MO, USA) and stopped with 1N H_2_SO_4_. Absorbance at 450 nm was measured using an ELISA plate reader (Tecan, Morrisville, NC, USA).

### 2.5. ZIKV Plaque-Forming Assay and Plaque Reduction Neutralization Test (PRNT)

Three recent ZIKV human strains, including R103451 (2015/Honduras), PAN2016 (2016/Panama), and PRVABC59 (2015/Puerto Rico), were used in the study. Briefly, viruses were grown in Vero E6 cells and detected for viral titers using a plaque-forming assay [27,32]. Mouse sera (about 50 µL) and tissues (about 20 mg for eye, and 40 mg for heart, spleen, muscle, and brain) collected 3 days post-challenge were also detected for ZIKV titers as described above, and the detection limits were about 20 plaque-forming unit (PFU)/mL for sera, 50 PFU/g for eye, or 25 PFU/g for heart, spleen, muscle, and brain tissues. Neutralizing antibodies in immunized mouse sera were detected by the PRNT as previously described [26,27]. Briefly, 100 PFU of ZIKV was incubated with 2-fold serial dilutions of mouse sera at 37 °C for 1.5 h, which were added to Vero E6 cells and incubated at 37 °C for 1 h. The cells were then overlaid with DMEM containing 1% carboxymethyl cellulose and 2% FBS, cultured at 37 °C for 4–5 days and further stained with 0.5% crystal violet. The neutralizing titer based on the serum neutralizations at a 50% plaque reduction (PRNT_50_) was calculated using the CalcuSyn computer program [27,33,34].

### 2.6. Challenge of Mice with ZIKV

The immunized mice were challenged with ZIKV as previously described [27,35]. Briefly, nine days post-last immunization of ZIKV EDIII protein with or without respective adjuvant(s), or PBS control, mice were pretreated with a blocking anti-interferon-α/β receptor 1 (Ifnar1) antibody (2 mg/mouse, Leinco Technologies, Fenton, MO, USA); and 24 h later, they were intraperitoneally (i.p.) challenged with ZIKV (strain R103451, 6 × 10^5^ PFU; 200 µL/mouse). Mouse splenocytes were isolated at 3 days post-challenge and detected for cellular immune responses using flow cytometry analysis. Mouse sera and tissues were collected as described above, and detected for ZIKV titers and RNA copies using the plaque-forming assay and quantitative reverse transcriptase PCR (qRT-PCR), respectively.

### 2.7. Flow Cytometry Analysis

Flow cytometry analysis was performed to detect ZIKV-specific cellular immune responses as previously described [29]. Briefly, splenocytes (2 × 10^6^ cells/well) were isolated from immunized mice 3 days post-ZIKV challenge, and treated with 1 × Red Blood Cell Lysis Buffer (Biolegend, San Diego, CA, USA), followed by incubation with a ZIKV EDIII peptide mixture (final concentration of 5 μg/mL) (Table 1) in the presence of brefeldin A (5 μg/mL, Biolegend), and cultured at 37 °C for 12 h. After stimulation, the cells were washed with PBS, and stained for surface markers using PerCP/Cy5.5 anti-mouse CD8-positive (CD8^+^), FITC-anti-mouse CD4-positive (CD4^+^), and AF700 anti-mouse CD45 antibodies (Biolegend). After fixation and permeabilization, the cells were further stained for intracellular markers using PE anti-mouse interferon-gamma (IFN-γ), Brilliant Violet 711^TM^ anti-mouse interleukin 4 (IL-4), and Brilliant Violet 605^TM^ anti-mouse IL-17 antibodies (Biolegend), which were analyzed using a flow cytometer (BD LSRFortessa 4 system).

### 2.8. qRT-PCR

This assay was performed to detect *ZIKV* RNA copies in sera and tissues of challenged mice as previously described [27,32]. Briefly, QIAamp MinElute Virus Spin Kit and RNeasy Mini Kit (Qiagen, Germantown, MD, USA) were used to extract RNAs from sera and tissues, respectively, which were quantified by qRT-PCR using Power SYBR Green PCR Master Mix, Ambion RNase Inhibitor, and MultiScribe Reverse Transcriptase (Thermo Fisher Scientific) in ViiA 7 Master Cycler PCR System (Thermo Fisher Scientific). The forward primer (5′-TTGGTCATGATACTGCTGATTGC-3′) and reverse primer (5′-CCTTCCACAAAGTCCCTATTGC-3′) were applied for amplification. A qRT-PCR standard curve was made via serial dilutions of a recombinant plasmid expressing the *ZIKV* membrane and E proteins, and a linear standard curve in the range of 10^1^–10^8^ RNA copies (correlation coefficient: R^2^ value > 0.98; detection limit: 10^1^ RNA copies) was selected for calculation of *ZIKV* RNA copies in the test samples. Mouse sera (about 10 µL) and tissues, including eye, heart, spleen (about 20 mg), muscle, and testis (about 40 mg), were collected 3 days post-challenge, and detected for *ZIKV* RNA, so the detection limit was about 10^3^ RNA copies/mL (for sera), 5 × 10^2^ RNA copies/g (for eye, heart, and spleen), and 2.5 × 10^2^ RNA copies/g (for muscle and testis) [32].

### 2.9. Statistical Analysis

All values were expressed as a mean with a standard error of the mean (s.e.m). Statistical significance of all data among different groups was calculated using one-way ANOVA: Tukey’s multiple comparison test based on the GraphPad Prism Statistical Software. *, **, and *** indicate *p* < 0.05, *p* < 0.01, and *p* < 0.001, respectively.

## 3. Results

### 3.1. Comparison of the Adjuvanticity of Alum, MPL, MF59, and in Combination, for Immunization with ZIKV EDIII

**Figure 1:**
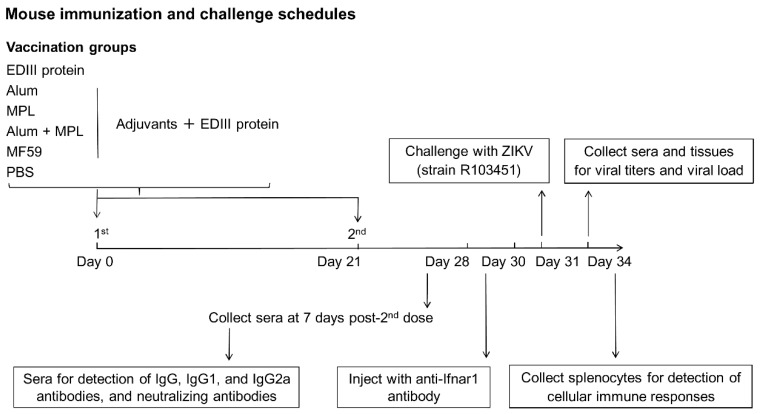
Immunization and challenge schedule. Mice were immunized intramuscularly (i.m.) with PBS (background control) or with ZIKV EDIII without an adjuvant, or with aluminum salts (Alum), monophosphoryl lipid A (MPL), Alum + MPL, or MF59 adjuvant and boosted at day 21 after the initial dose. Sera were collected at seven days after the boost (day 28) and used to determine ZIKV E or EDIII-specific antibody, and neutralizing antibody levels. Mice were injected with an anti-interferon-α/β receptor 1 (Ifnar1) antibody (in order to become susceptible to subsequent ZIKV infection) [36,37] at nine days after the boost (day 30) and subsequently challenged with ZIKV (strain R103451, 6 × 10^5^ plaque-forming unit (PFU)) the following day (day 31). Sera and tissue samples were collected three days post-challenge (day 34) and used to determine ZIKV titers and viral load (RNA copies). Splenocytes were also tested for the ZIKV EDIII-specific cellular immune response.

**Figure 2:**
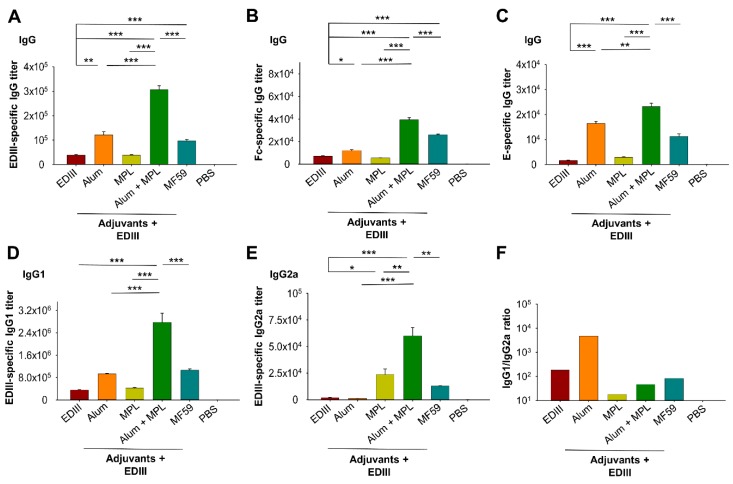
Detection of specific antibodies in mouse sera. ZIKV EDIII-, E-, or Fc of human IgG1 (hFc)-specific antibody levels were determined by ELISA using sera collected at seven days following the second dose of ZIKV EDIII. (**A**) ZIKV EDIII-specific IgG; (**B**) hFc-specific IgG; (**C**) ZIKV E-specific IgG; (**D**) ZIKV EDIII-specific IgG1; (**E**) ZIKV EDIII-specific IgG2a antibodies. (**F**) The IgG1/IgG2a ratio for EDIII without or with each adjuvant, or adjuvant combination. ELISA plates were coated with ZIKV EDIII containing a C-terminal hFc or full-length E protein containing a His_6_ tag. Middle East respiratory syndrome coronavirus (MERS-CoV) receptor-binding domain (RBD)-Fc protein (i.e., MERS-CoV RBD containing a C-terminal hFc) was used to test hFc-specific IgG antibodies. Adjuvants are indicated in all the figures. PBS represents the background level of specific antibodies. The antibody titers shown are the endpoint dilution where a positive signal was detected. Data are shown as the mean ± the standard error of the mean (s.e.m) (*n* = 5). Significant differences (*: *p* < 0.05; **: *p* < 0.01; ***: *p* < 0.001) among different groups are shown.

To compare the potency of adjuvants, BALB/c mice were immunized with a ZIKV non-neutralizing epitope-masked, EDIII subunit vaccine in the presence or absence of Alum, MPL, and Alum adjuvant in combination with the MPL adjuvant. In addition, MF59 was included as a control adjuvant. At seven days following the second dose, sera were collected and the antibody titers specific to ZIKV EDIII (containing a C-terminal hFc tag) or E (without fusion with hFc) were determined (Figure 1). When used without an adjuvant, EDIII was immunogenic and induced EDIII-specific IgG antibodies (total IgG), while inclusion of Alum or MF59 adjuvant significantly improved the antibody response. Although MPL did not appear to enhance the antibody response to EDIII, when used in combination with Alum, the antibody response was significantly higher than for EDIII with Alum or MF59 alone (Figure 2A). Since EDIII protein was fused with the hFc tag, IgG antibodies targeting hFc were also generated, showing a similar trend as those specific to EDIII (Figure 2B). Nevertheless, when coating the ELISA plate with a ZIKV full-length E protein without hFc, significantly high-titer IgG antibodies were induced, particularly in the Alum and MPL-adjuvanted EDIII (Figure 2C), suggesting that fusion of hFc to the EDIII subunit vaccine did not affect the generation of ZIKV-specific IgG antibodies.

We also determined the levels of the IgG1 and IgG2a antibodies induced by EDIII at seven days after the second dose. EDIII alone induced a good IgG1 response while the IgG2a response was noticeably weaker (Figure 2D,E). When used alone, Alum induced higher levels of IgG1 (but not IgG2a) antibodies than EDIII without an adjuvant, while MPL induced significantly higher levels of IgG2a (but not IgG1) antibodies. When used in combination, Alum with MPL significantly improved the ability of EDIII to induce the IgG1 and IgG2a antibody responses (Figure 2D,E). Moreover, the combination of Alum with MPL adjuvant induced significantly higher IgG1 and IgG2a antibody titers than either Alum or MPL alone, or when MF59 was the adjuvant (Figure 2D,E). When used in combination, MPL and Alum adjuvants induced a more balanced antibody response, indicated by a low IgG1 (Th2)/IgG2a (Th1) ratio. In contrast, Alum generated a Th2-biased IgG1 antibody response, while EDIII alone, or when formulated with MF59, showed a slight Th2 bias (Figure 2F). The above data demonstrate that the combination of Alum with MPL adjuvant enhanced the levels of EDIII-specific IgG antibodies (IgG1 and IgG2a) and produced a more balanced IgG response.

### 3.2. The Effect of Alum in Combination with MPL Adjuvant on Anti-ZIKV Neutralizing Antibody Titers

**Figure 3:**
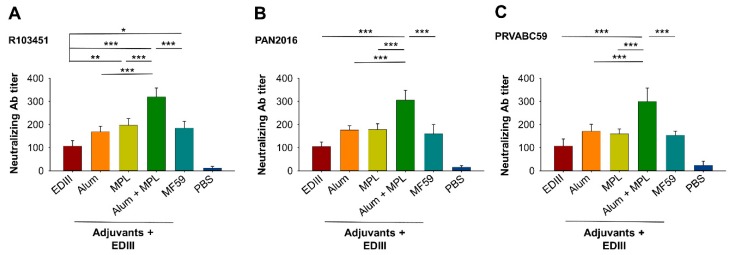
Detection of neutralizing antibody titers in immunized mouse sera. Sera collected at seven days following the second dose of EDIII were tested by a plaque reduction neutralization test (PRNT) assay for neutralizing antibodies against ZIKV human strains R103451 (**A**), PAN2016 (**B**), and PRVABC59 (**C**). PBS shows the background level of neutralizing antibodies. Neutralizing activity for the indicated adjuvants is presented as the neutralizing antibody titer based on the serum neutralizations at a 50% plaque reduction (PRNT_50_). Data are expressed as the mean ± s.e.m (*n* = 5). Significant differences (*: *p* < 0.05; **: *p* < 0.01; ***: *p* < 0.001) among different groups are shown.

Next, we compared the levels of anti-ZIKV neutralizing antibodies induced by EDIII vaccine when formulated with the different adjuvants. To this end, sera collected at seven days after immunization with the second dose of EDIII were tested for neutralization against three human ZIKV strains (R103451, PAN2016, and PAVABC59). For all three strains, Alum and MPL as the adjuvants induced a significantly stronger neutralizing antibody response than EDIII alone, while MPL or MF59 only enhanced the neutralizing antibody response for the R103451 strain (Figure 3A–C). Importantly, for all three ZIKV strains, the neutralizing antibody titers induced by EDIII were significantly higher when Alum was used in combination with MPL than for Alum, MPL, or MF59 as the sole adjuvant (Figure 3A–C). In contrast, sera injected with PBS only induced a background-level neutralizing antibody (Figure 3A–C). These data show that although single adjuvants increased the anti-ZIKV neutralizing antibody response, the combination of Alum with MPL adjuvant consistently induced the highest level of neutralizing antibodies against all of the ZIKV strains investigated.

### 3.3. The Effect of Alum in Combination with MPL Adjuvant on the ZIKV EDIII-Specific Cellular Immune Response

**Figure 4:**
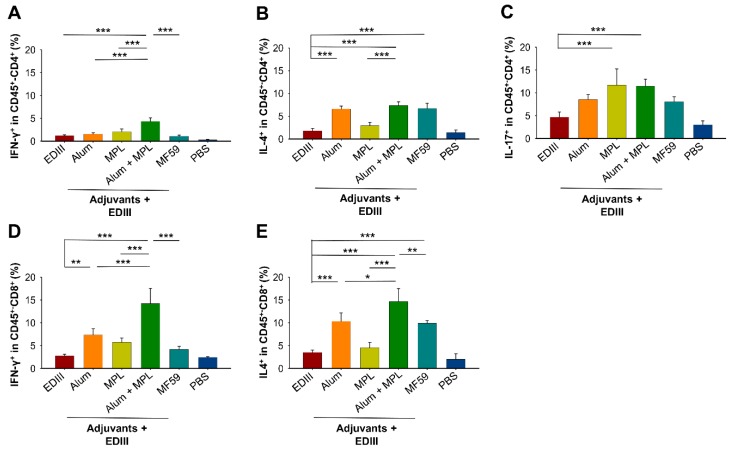
The effect of Alum and MPL adjuvants on the ZIKV EDIII-specific cellular immune response. Splenocytes were isolated at three days post-challenge with ZIKV (strain R103451) and used to determine secretion of IFN-γ (**A**), IL-4 (**B**), and IL-17 (**C**) by CD45-positive (CD45^+^)–CD4-positive (CD4^+^) T cells, and of IFN-γ (**D**) and IL-4 (**E**) by CD45^+^–CD8-positive (CD8^+^) T cells using flow cytometry analysis. PBS shows the background level of cytokines. Data are expressed as the mean ± s.e.m (*n* = 5). Significant differences (*: *p* < 0.05; **: *p* < 0.01; ***: *p* < 0.001) among different groups are shown.

Splenocytes isolated from immunized mice three days after ZIKV challenge (strain R103451) were used to investigate if the adjuvants enhanced the ZIKV EDIII-specific cellular immune response. Specifically, we determined the levels of secretion of IFN-γ (Th1), IL-4 (Th2) and IL-17 (Th17) by CD45-positive (CD45^+^)–CD4^+^ T cells, and IFN-γ and IL-4 by CD45^+^–CD8^+^ T cells. For both T cell subsets, only low levels of these cytokines were detected when mice were immunized with EDIII alone (Figure 4A–E), suggesting that EDIII without an adjuvant did not induce a strong EDIII-specific T cell response. When used individually, Alum or MF59 increased the secretion of IL-4 by CD45^+^–CD4^+^ T cells, while increased expression of IL-17 was only seen for MPL (Figure 4B,C). For CD45^+^–CD8^+^ T cells, inclusion of Alum significantly increased IFN-γ expression, while IL-4 expression was increased by Alum and MF59 (Figure 4D,E). When used in combination, Alum with MPL adjuvant significantly enhanced expression of IFN-γ and IL-4 cytokines tested in both CD45^+^–CD4^+^ and CD45^+^–CD8^+^ T cells over that observed when the adjuvants (Alum, MPL, and/or MF59) were used individually (Figure 4A,B,D,E). These data indicate that although individual adjuvants enhanced the EDIII-specific cellular immune response, the combination of Alum with MPL had a greater effect, particularly in CD45^+^–CD8^+^ T cells.

### 3.4. The Effect of Alum in Combination with MPL Adjuvant on Protective Efficacy of ZIKV EDIII Vaccine, Including Reproductive Organs

**Figure 5:**
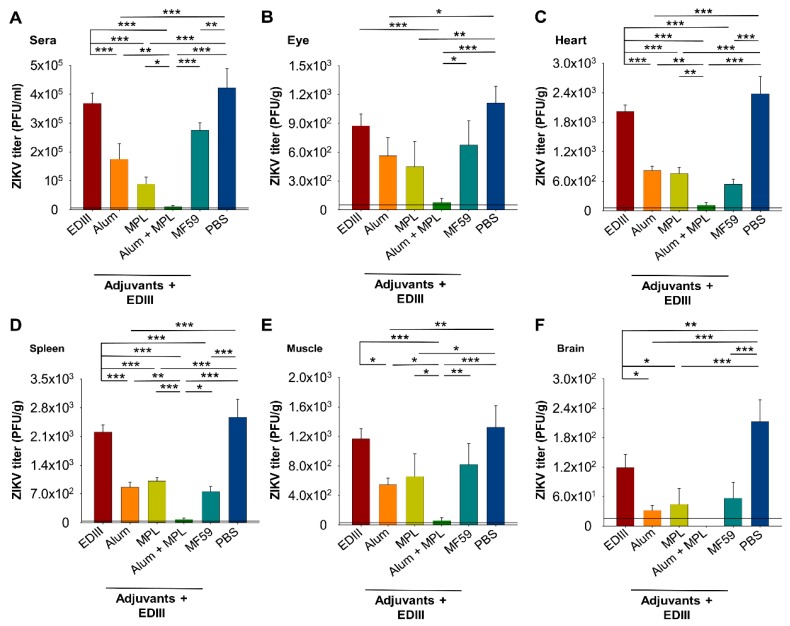
Detection of ZIKV titers in challenged sera (**A**), eye (**B**), heart (**C**), spleen (**D**), muscle (**E**), and brain (**F**). Sera and tissues were collected at three days post-challenge with ZIKV (strain R103451), and viral load determined by a plaque-forming assay. Mice immunized with PBS served as the control. The detection limits for the samples were ~20 PFU/mL (sera), ~50 PFU/g (eye), and ~25 PFU/g (heart, spleen, muscle, and brain). Data are expressed as the mean ± s.e.m (*n* = 5). Significant differences (*: *p* < 0.05; **: *p* < 0.01; ***: *p* < 0.001) among different groups are shown.

To investigate if the adjuvants increased protection against ZIKV infection, immunized mice were challenged with a high-dose ZIKV (strain R103451, 6 × 10^5^ PFU/mouse) one day after administering an anti-Ifnar1 antibody (Figure 1). Viral load was determined by a plaque-forming assay and a *ZIKV* RNA copy number determined by qRT-PCR assay using sera and tissues collected three days post-challenge, an optimal time point to detect ZIKV infection in anti-Ifnar1 antibody-treated immunocompetent mice, including BALB/c (our unpublished data) [37]. Similar levels of ZIKV were seen in sera and most tissues (eye, heart, spleen, and muscle) for mice immunized with EDIII without an adjuvant or with PBS (Figure 5A–E), whereas only in brain tissue, a significant reduction in viral titers was observed for mice immunized with EDIII alone (Figure 5F). These results suggest that immunization with EDIII is unable to elicit adequate protection against ZIKV challenge. Mice immunized with EDIII formulated with a single adjuvant (Alum, MPL, and/or MF59) had significantly reduced viral titers compared to those immunized with EDIII alone (sera, heart, spleen, muscle, and brain) (Figure 5A,C–F) and those immunized with PBS (sera, eye, heart, spleen, muscle, and brain) (Figure 5A–F), suggesting that inclusion of an adjuvant increased protection. Importantly, in sera and all of the tissues tested, the combination of Alum with MPL adjuvant resulted in significantly lower, or undetectable, viral titers than other immunization conditions with a single adjuvant (Alum, MPL, and/or MF59) (Figure 5A–F). These results demonstrate that, although single adjuvants can increase protection, the greatest level of protection against a high-dose ZIKV challenge is achieved when EDIII vaccine is formulated with both Alum and MPL adjuvants.

**Figure 6:**
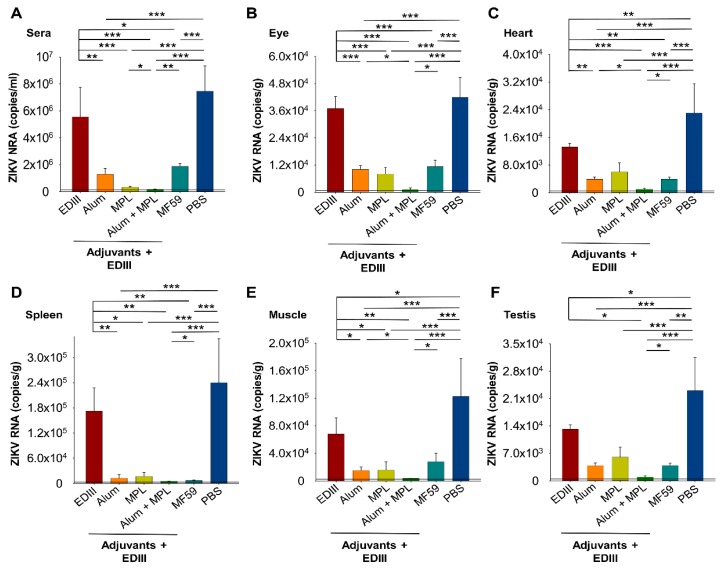
Detection of ZIKV RNA in challenged sera (**A**), eye (**B**), heart (**C**), spleen (**D**), muscle (**E**), and testis (**F**). Sera and tissues were collected at three days post-challenge with ZIKV (strain R103451) and viral RNA copies determined by qRT-PCR. Mice immunized with PBS served as the control. The detection limits were ~10^3^ RNA copies/mL (sera), ~5 × 10^2^ RNA copies/g (eye, heart, and spleen), and ~2.5 × 10^2^ RNA copies/g (muscle and testis). Data are expressed as the mean ± s.e.m (*n* = 5). Significant differences (*: *p* < 0.05; **: *p* < 0.01; ***: *p* < 0.001) among different groups are shown.

Consistent with viral load, mice immunized with EDIII alone had only slightly lower levels of *ZIKV* RNA in sera and tissues (eye and spleen) compared to those immunized with PBS (Figure 6A,B,D), confirming that an adjuvant is required to induce protection. Similarly, inclusion of a single adjuvant (Alum, MPL, and/or MF59) resulted in a significantly lower level of *ZIKV* RNA in sera and tissues (eye, heart, spleen, or muscle) compared with immunization with EDIII alone (Figure 6A–E). Furthermore, the levels of *ZIKV* RNA in sera and all of the tissues, when Alum and MPL adjuvants were used in combination for immunization, were significantly lower than when a single adjuvant (Alum, MPL, and/or MF59) was used (Figure 6A–E). These results are consistent with the levels of viral load (Figure 5) and confirm that protection against ZIKV is greatest when Alum and MPL adjuvants are used in combination.

ZIKV can replicate in the reproductive organs for long periods; therefore, we also determined the amount of *ZIKV* RNA in testes of immunized and challenged mice. Similar to the results in other tissues, inclusion of a single adjuvant reduced the level of viral RNA more than immunization with EDIII alone or with PBS (Figure 6F). Moreover, viral RNA copies were the lowest in testis of mice receiving EDIII formulated with Alum and MPL in combination (Figure 6F). Importantly, our results show that formulation of EDIII with a combination of Alum and MPL adjuvants can reduce viral RNA copies in male reproductive organs.

## 4. Discussion

Protein-based subunit vaccines are generally safer than vaccines based on live attenuated or inactivated viruses but usually have low immunogenicity and efficacy [20,21]. However, vaccine performance can be improved by structure-based designs via masking non-neutralizing or unfavorable epitopes, or via antigen conjugation [27,38,39]. In addition, the choice of adjuvant can greatly influence the immunogenicity and efficacy of vaccines, including subunit vaccines [29,40,41].

Our previous studies demonstrated that masking of a non-neutralizing epitope (surrounding residue 375) of a ZIKV DIII of E protein subunit vaccine (i.e., EDIII) improved immunogenicity and protection of adult and pregnant mice, and unborn offspring, against ZIKV infection [27]. Here, we extended these studies to investigate the influence of licensed adjuvants on the immunogenicity and protection of mice against high-dose ZIKV challenge following immunization with this EDIII subunit vaccine. Vaccines prepared with a single adjuvant (Alum, MPL, or MF59) significantly improved the antibody response (total IgG, IgG1, and/or IgG2a) over immunization without an adjuvant. However, the highest IgG titers were obtained when Alum and MPL were used in combination.

As stated above, adjuvants influence the immune response to vaccines. Alum is a potent inducer of the Th2 (IgG1) immune response [23,42]. Similarly, MF59 generally induces a slightly-biased Th2 response as seen for vaccines for influenza virus and MERS-CoV [29,43]. In contrast, MPL induces expression of IFN-γ and IL-2 and, therefore, has a more Th1 bias and generates a more balanced (Th1/Th2) immune response [22,25]. Moreover, a combination of MPL and Alum adjuvants (i.e., AS04) may shift the immune responses from Th2 to Th1 and generate a more balanced immune response [44]. Consistent with these observations, we found that MF59 and Alum, when used individually as the adjuvant for EDIII, induced a Th2-biased immune response, while, when MPL was the sole adjuvant or used in combination with Alum, a more balanced (Th1/Th2) immune response was generated.

The humoral and cellular immune responses influence ZIKV pathogenesis and play key roles in controlling ZIKV replication [45]. In particular, neutralizing antibodies, induced by ZIKV vaccines, may block infection [27,46], while CD4^+^ and CD8^+^ T cells secrete IFN-γ, IL2 (Th1), IL-4, and IL-5 (Th2) cytokines to further promote antibody production or directly kill infected cells [47,48,49,50]. CD8^+^ T cells are critical for protection against ZIKV infection in mice, non-human primates, and humans [45,48,50,51,52]. In the current study, we observed that Alum, MPL, and MF59 adjuvants enhanced production of anti-ZIKV neutralizing antibodies; however, the combination of Alum with MPL generated significantly higher neutralizing antibody titers than the individual use of adjuvants, and these results were consistent for three emerging human ZIKV strains. In addition, Alum and MPL, when used in combination, enhanced secretion of IFN-γ (Th1) and IL-4 (Th2) by CD4^+^, particularly CD8^+^, T cells. Viral vaccines (e.g., against HBV and HPV) formulated with a combination of Alum and MPL adjuvants (i.e., AS04) stimulate antigen-specific T and B cells, resulting in stronger antibody (including neutralizing antibodies) and cellular responses than vaccines formulated using Alum alone [22,44]. Using the ZIKV EDIII protein as a model antigen, our study has confirmed these observations by showing that a combination of Alum and MPL adjuvants enhanced the immunogenicity of EDIII over that observed with single adjuvants (Figure 2, Figure 3, Figure 4, Figure 5 and Figure 6), whereas the Alum and MPL combination alone without EDIII was unable to elicit ZIKV-specific immune response and protection against ZIKV infection [26,27].

ZIKV does not normally infect immunocompetent mice, including BALB/c and C57BL/6 [36,53], but does infect immunocompromised mice deficient for interferon receptors (interferon-α/β receptor (Ifnar) or A129, or interferon-α/β/γ receptor, AG129) [36,53,54]. To carry out challenge studies, we used BALB/c mice treated with anti-Ifnar1 antibodies, which also render mice susceptible to ZIKV infection [36,37]. Formulation of EDIII with individual adjuvants improved protection against high-dose ZIKV challenge, and was consistent with our virus neutralization studies results, that is, the combination of Alum with MPL significantly improved protection further. This was confirmed by showing that a combination of Alum with MPL as the adjuvant significantly reduced viral titers and viral RNA copies in sera and tissues compared to other immunization regimes. ZIKV can be transmitted through sexual contact, the virus can persist in female or male (testes and seminal vesicles) reproductive organs for a prolonged period, and viral RNA can be detected in semen for up to one year after ZIKV infection [53,55,56,57,58,59]. Thus, vaccines that reduce viral load in reproductive organs will likely be more effective in reducing virus transmission during sexual contact. Importantly, our results show that EDIII formulation using Alum and MPL in combination significantly reduced viral RNA in the testes of ZIKV-challenged mice, thereby confirming that this adjuvant combination is most likely to yield greater vaccine efficacy than single adjuvants alone. In contrast, EDIII alone without adjuvant(s) resulted in the highest viral titers and viral RNA copies in sera and tissues tested among all vaccination groups, including reproductive organs, which were only slightly lower than those in PBS-injected control mice. It has been shown that protection against ZIKV infection is positively correlated with serum neutralizing antibody titers [26,27]; thus, the high-level ZIKV in the EDIII alone-immunized mice identified above may be partially due to the relatively low serum neutralizing antibody titer induced in these mice.

It should be noted that the EDIII protein used in this study was fused with a C-terminal hFc tag. Fc-fusion proteins have been approved by the FDA for human therapy, and their safety and pharmacokinetic activity have been evaluated extensively [60,61,62,63]. Fusion of Fc tag to recombinant proteins may promote the proteins to form dimeric structures, significantly improving their solubility, stability, and avidity, as well as increasing their immunogenicity and/or efficacy [64,65,66]. Fc-fused vaccine design has been applied in subunit vaccines against HIV, influenza virus, Ebola virus, and other emerging or reemerging viruses [66,67,68,69,70]. In terms of ZIKV vaccines, a Fc-fused E protein dimer (ZE-Fc) has been shown to mimic native dimeric status of E protein and induce higher neutralizing antibodies than a monomeric E protein without Fc (monZE) [71]. We have also found that hFc-fused ZIKV EDIII proteins form dimeric structures capable of eliciting high-titer antibody responses, neutralizing antibodies, and protection against high-dose ZIKV infection (Figure 2, Figure 3, Figure 5 and Figure 6) [27], consistent with previous findings.

In conclusion, using immunocompetent BALB/c mice, we compared the immunogenicity, neutralizing activity, and protective efficacy (after injection with anti-Ifnar1 antibodies) of a ZIKV EDIII subunit vaccine when formulated with different adjuvants (individually or in combination). Although EDIII formulated with Alum, MPL, or MF59 induced a specific antibody and cellular immune response, and reduced viral load, the greatest vaccine efficacy was achieved when Alum and MPL adjuvants were combined in a single vaccine. Overall, this study has identified Alum combined with MPL as the adjuvant of choice for ZIKV subunit vaccines, and has important implications for subunit vaccines against other enveloped viruses, including non-ZIKV flaviviruses.

## Figures and Tables

**Table 1:** Zika virus (ZIKV) DIII of E (EDIII) protein peptides used for stimulation of cellular immune responses.

Peptide Name	Sequence	Peptide Name	Sequence
1	LRLKGVSYSLCTAAF	2	TFTKIPAETLHGTVT
3	VELQYAGTDGPCKVP	4	AQMAVDMQTLTPVGR
5	LITANPVITESTENS	6	KMMLELDPPFGDSYI
7	VIGVGEKKITHHWHR	8	THHWHRSGSTIGKAF
